# The Adenovirus E4orf4 Protein Provides a Novel Mechanism for Inhibition of the DNA Damage Response

**DOI:** 10.1371/journal.ppat.1005420

**Published:** 2016-02-11

**Authors:** Anna Brestovitsky, Keren Nebenzahl-Sharon, Peter Kechker, Rakefet Sharf, Tamar Kleinberger

**Affiliations:** Department of Microbiology, the Rappaport Faculty of Medicine and Research Institute, Technion–Israel Institute of Technology, Haifa, Israel; Wake Forest University, UNITED STATES

## Abstract

The DNA damage response (DDR) is a conglomerate of pathways designed to detect DNA damage and signal its presence to cell cycle checkpoints and to the repair machinery, allowing the cell to pause and mend the damage, or if the damage is too severe, to trigger apoptosis or senescence. Various DDR branches are regulated by kinases of the phosphatidylinositol 3-kinase-like protein kinase family, including ataxia-telangiectasia mutated (ATM) and ATM- and Rad3-related (ATR). Replication intermediates and linear double-stranded genomes of DNA viruses are perceived by the cell as DNA damage and activate the DDR. If allowed to operate, the DDR will stimulate ligation of viral genomes and will inhibit virus replication. To prevent this outcome, many DNA viruses evolved ways to limit the DDR. As part of its attack on the DDR, adenovirus utilizes various viral proteins to cause degradation of DDR proteins and to sequester the MRN damage sensor outside virus replication centers. Here we show that adenovirus evolved yet another novel mechanism to inhibit the DDR. The E4orf4 protein, together with its cellular partner PP2A, reduces phosphorylation of ATM and ATR substrates in virus-infected cells and in cells treated with DNA damaging drugs, and causes accumulation of damaged DNA in the drug-treated cells. ATM and ATR are not mutually required for inhibition of their signaling pathways by E4orf4. ATM and ATR deficiency as well as E4orf4 expression enhance infection efficiency. Furthermore, E4orf4, previously reported to induce cancer-specific cell death when expressed alone, sensitizes cells to killing by sub-lethal concentrations of DNA damaging drugs, likely because it inhibits DNA damage repair. These findings provide one explanation for the cancer-specificity of E4orf4-induced cell death as many cancers have DDR deficiencies leading to increased reliance on the remaining intact DDR pathways and to enhanced susceptibility to DDR inhibitors such as E4orf4. Thus DDR inhibition by E4orf4 contributes both to the efficiency of adenovirus replication and to the ability of E4orf4 to kill cancer cells.

## Author Summary

The cellular DNA damage response (DDR) network interprets the presence of replicating viral DNA genomes as DNA damage and strives to repair it, leading to inhibition of virus replication. Many DNA viruses, including adenovirus, evolved mechanisms to inhibit the DDR, thus increasing the efficiency of virus replication. In this study we identify a novel mechanism used by adenovirus to inhibit the DDR. The viral E4orf4 protein, together with its cellular partner, the PP2A phosphatase, inhibits damage signaling by reducing phosphorylation of proteins belonging to different DDR branches. As a result, E4orf4 causes accumulation of DNA damage in the cells. Inhibition of the DDR regulators ATM and ATR, as well as expression of E4orf4, enhances infection efficiency. Moreover, E4orf4 sensitizes cells to killing by sub-lethal concentrations of DNA damaging drugs, likely because it inhibits DNA repair. These findings could provide one explanation for the previously reported ability of E4orf4 to induce cancer-specific cell death, as many cancers have DDR deficiencies leading to their increased reliance on the remaining intact DDR pathways and to enhanced susceptibility to DDR inhibitors such as E4orf4. Thus, inhibition of the DDR by E4orf4 contributes both to viral replication efficiency and to E4orf4-induced cancer cell killing.

## Introduction

Genome integrity is constantly challenged by endogenous and exogenous agents that cause different kinds of DNA lesions. The cells have evolved a DNA damage response (DDR) which includes several mechanisms to detect and signal the presence of damaged DNA or replication stress, resulting in checkpoint activation and DNA repair, or if the damage is too extensive, resulting in senescence or cell death [1, 2]. Formation of DNA lesions is recognized by sensor proteins such as Poly (ADP-ribose) polymerase 1 (PARP-1) [3, 4], KU proteins [5], or the MRN complex consisting of the Mre11, Rad50 and Nbs1 proteins [6–9]. The sensors recruit proteins that transduce the signal to chromatin, to cellular checkpoints and to the repair machinery [10]. Signal transducers include the phosphatidylinositol 3-kinase-like protein kinase (PIKK) family, including ataxia-telangiectasia mutated (ATM), ATM- and Rad3-related (ATR), and DNA-PK (reviewed in [11, 12]).

Protein phosphatase 2A (PP2A) is composed of three subunits: the catalytic C subunit, a scaffolding A subunit, and one of several regulatory B subunits encoded by at least four unrelated gene families: PR55/B55/B, PR61/B56/B’, B”, and B”‘[13], which dictate substrate specificity of the PP2A holoenzyme [13]. PP2A plays an important role in the DDR by regulating the activity of PIKKs [14–18] and their substrates Chk1, Chk2, or γH2AX, which are part of the DDR signaling pathways [19–24].

During infection with DNA viruses, stretches of single-stranded DNA in replication intermediates and the ends of the double stranded viral DNA are perceived by the cell as damaged DNA, inducing the DDR (reviewed in [25, 26]). Some viruses may exploit the DDR to their advantage [27–29], however, the end result of DDR activation may lead to repair of viral genomes through their concatenation [30–32] and is detrimental to virus propagation. Therefore, many viruses, including adenovirus (Ad), have evolved mechanisms to inhibit the DDR. It has been previously reported that various types of Ad inhibit the DDR by ubiquitin-mediated degradation of DDR proteins including components of the MRN complex [6, 31, 33], DNA ligase IV [33, 34], p53 [33, 35, 36], TOPBP1 [33, 37], and others, or by removing MRN from viral replication foci [31, 38, 39]. These Ad effects are promoted by the E4orf3, E4orf6, and E1B-55K proteins. The cellular response to Ad genomes was recently reported to be biphasic, including a MRN-ATM-dependent DDR that is activated by early replicating virus genomes, is localized at viral replication centers and must be inactivated by the E1B-55K and E4orf3 proteins to allow viral replication; and a global MRN-independent ATM activation induced by viral nuclear domains that does not affect virus replication [40]. At early times after infection, the incoming viral genome is protected from the DDR by the adenoviral core protein VII [41]. The E4orf6 protein was also reported to inhibit double strand breaks (DSB) repair signaling through inhibition of PP2A leading to prolonged presence of γH2AX and PARP hyperactivation and resulting in enhanced apoptosis [42].

The Ad E4orf4 protein is a multifunctional viral regulator. Within the context of the virus E4orf4 contributes to temporal regulation of the progression of viral infection by down-regulating early viral genes and cellular genes affecting Ad replication, controlling alternative splicing of viral RNAs, and influencing protein translation [43–50]. When expressed alone E4orf4 induces cell death which is p53- and caspase-independent but frequently maintains crosstalk with classical caspase-dependent apoptosis [51–54]. The E4orf4 signaling network is highly conserved in evolution from yeast through *Drosophila* to mammalian cells [55–59], underscoring its importance to cell regulation. Moreover, E4orf4-induced cell death is more efficient in oncogene-transformed cells in tissue culture than in normal cells [60], indicating that study of E4orf4 signaling may have implications for cancer therapy. The basis for the cancer specificity of E4orf4-induced cell death will be better understood once knowledge of the underlying mechanisms is improved [61].

Studies of the mechanisms underlying E4orf4 action have revealed several E4orf4 partners, including the B55 subunit of protein phosphatase 2A (PP2A) [62]. PP2A is a major E4orf4 partner, and its interaction with E4orf4 was shown to contribute to all E4orf4 functions known to date [50, 60–64].

Here we report that E4orf4 provides a novel mechanism to inhibit DNA damage signaling during Ad infection. We show that E4orf4 expression leads to decreased phosphorylation of several proteins that participate in the DDR, in both ATM- and ATR-regulated pathways and causes accumulation of DNA damage. This novel E4orf4 function requires its interaction with the B55 subunit of PP2A. Inhibition of the DDR by E4orf4 has biological significance to Ad infection and to E4orf4-induced cell death.

## Results

### E4orf4 inhibits DNA damage signaling during mutant Ad infection

**Fig 1:**
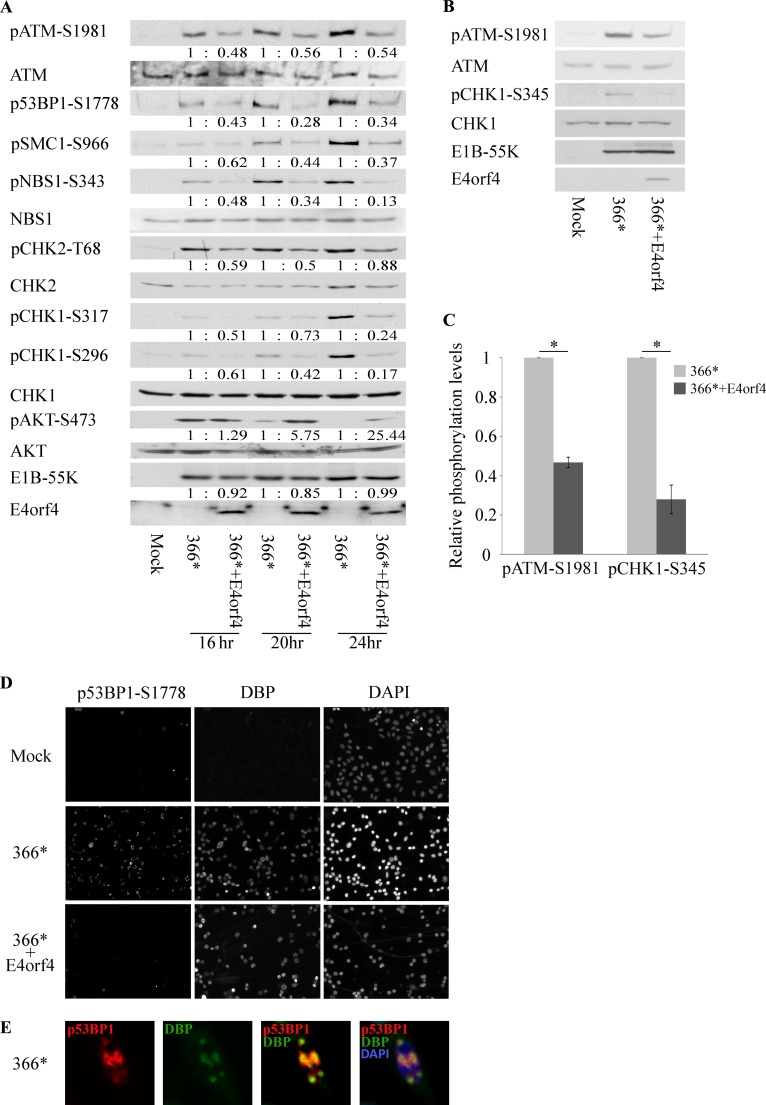
E4orf4 reduces the phosphorylation of DDR proteins during mutant Ad infection. (**A**) HeLa cells were either mock-infected or infected with the Ad mutant dl366*, lacking the E4 region, or with dl366*+E4orf4, lacking all E4 orfs except E4orf4. Infections were done with 20 ffu per cell. Proteins were harvested at the indicated times post infection and Western blot analysis was carried out with the specified antibodies. Protein bands were quantified by densitometry, the levels of phosphorylated proteins were normalized to the levels of the corresponding total proteins, and the ratios between normalized phosphorylation in dl366*-infected cells (defined as 1) and parallel values in dl366*+E4orf4-infected cells are shown below the bands. (**B**) HUVEC cells were either mock-infected or infected with the Ad mutants dl366* and dl366*+E4orf4 at 30 ffu per cell. Proteins were harvested 24 hrs post infection and Western blot analysis was carried out with the specified antibodies. (**C**) The intensities of protein bands in Western blots from two independent experiments performed as described in B were quantified by densitometry. Relative phosphorylation levels were determined as described in A and the average of the two experiments is shown in the graph. Error bars represent standard error. *: p<0.005. (**D**) HeLa cells were infected as in A, fixed 24 hrs post infection, and stained with the indicated antibodies and with DAPI. Representative pictures taken with a Zeiss Axioskop at a magnification of 100 are shown. (**E**) A representative picture of a dl366*-infected HeLa cell stained with antibodies to p53BP1-S1778 and the Ad DNA binding protein (DBP) and with DAPI is shown at a magnification of 640.

It has been reported that Ad interferes with the DDR as part of its protection against host defense mechanisms, using the E1B-55K, E4orf6, and E4orf3 viral proteins [6, 31, 33, 39]. Since viruses usually use several different mechanisms to achieve a single aim, and since it has been shown that PP2A participates in turning off the DDR by dephosphorylating DDR proteins such as ATM, Chk1, and Chk2 [15, 17, 20–23], we set out to determine whether the Ad E4orf4 protein, a PP2A partner, contributes to down-regulation of virus-induced DDR. HeLa cells were either mock-infected or infected with the Ad mutant dl366*, lacking the E4 region, or with dl366*+E4orf4, lacking all E4 orfs except E4orf4 [65]. These mutants were used to facilitate the examination of E4orf4 effects when the MRN complex is unharmed by other E4 gene products and the DDR is activated [6, 31, 33, 39]. Protein extracts were prepared at various times after infection and subjected to Western blot analysis. The uniformity of infection was confirmed by finding similar levels of the E1B-55K protein in cells infected with the two E4 mutant viruses (Fig 1A). It should be noted that the E4orf4 protein was reported to reduce the levels of early Ad mRNAs in HeLa cells [44, 45], but E1B levels were affected less than those of other early RNAs [44]. Fig 1A demonstrates that infection with the mutant dl366* led to phosphorylation of several DDR proteins, including ATM, 53BP1, Smc1, Nbs1, Chk1, and Chk2, while these proteins were not phosphorylated in mock-infected cells. In contrast, infection with a similar multiplicity of infection (MOI) of dl366*+E4orf4 mutant Ad resulted in decreased phosphorylation levels of all tested DDR proteins belonging to both ATM- and ATR-regulated pathways. The reduced phosphorylation was confirmed by quantification of the protein bands and normalization to total protein levels. The E4orf4-induced decrease in DDR protein phosphorylation did not represent a global reduction of cellular phosphorylation as E4orf4 did not reduce Akt-S473 phosphorylation, but rather enhanced it. Thus the combined results suggest that E4orf4 reduces DDR induction by an E4-deficient mutant Ad. This conclusion was further validated by similar experiments carried out in non-transformed, primary HUVEC cells (Fig 1B and 1C), indicating that E4orf4 inhibited the DDR in both transformed and non-transformed cells. It should be noted that although phosphorylated H2AX (γH2AX) is a characteristic marker of DSBs, WT Ad infection was reported to result in high levels of γH2AX [66], despite the degradation of the MRN sensor complex and the reduced phosphorylation of downstream substrates. We therefore did not investigate γH2AX in the presence of E4orf4.

To further confirm the results indicating that E4orf4 reduced DDR protein phosphorylation, HeLa cells were infected with mutant viruses as described above and subjected to immunofluorescence staining with antibodies to the 53BP1 protein phosphorylated on S1778. Infected cells were identified by staining of the Ad DBP protein. Fig 1D demonstrates that cells infected with dl366* contained foci of phosphorylated 53BP1 in 75% of infected cells and these p53BP1-S1778-stained foci surrounded nuclear viral compartments in the infected cells (Fig 1E), similarly to other DDR proteins described previously [6, 31, 40]. In contrast, infection with dl366*+E4orf4 did not result in accumulation of phospho-53BP1 and foci containing p53BP1-S1778 were observed in only 6% of infected cells. Thus the results presented in Fig 1 support the conclusion that E4orf4 inhibits DNA damage signaling.

### DDR inhibition by E4orf4 is PP2A-dependent

**Fig 2:**
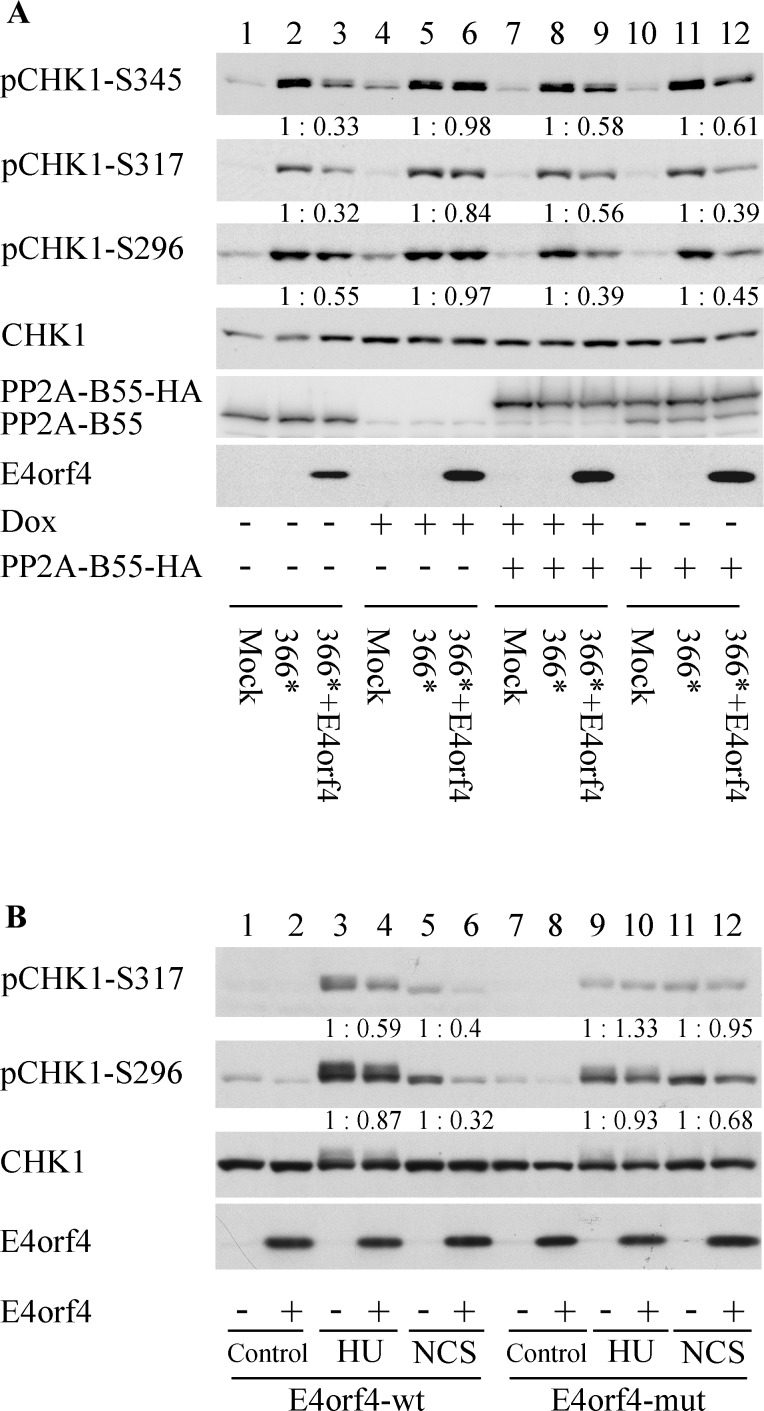
DDR inhibition by E4orf4 is PP2A-dependent. (**A**) L11 cells in which a PP2A-B55 shRNA can be transiently expressed following Dox induction were induced with Dox (+) or left untreated (-). 48 hrs later, the cells were transfected with an empty vector (-), or with a vector expressing shRNA-resistant PP2A-B55-HA (+). One day later, dl366* and dl366*+E4orf4 mutant viruses were used to infect the transfected cells, and some of the cells were left uninfected (Mock). Protein extracts were prepared 24 hrs post-infection and subjected to Western blot analysis with the indicated antibodies. Quantitation and normalization were performed as described in the legend to Fig 1. (**B**) HEK293-derived clone 13 cells containing Dox-inducible WT E4orf4 or clone 3 cells expressing an E4orf4 mutant unable to bind PP2A (R81F84A: E4orf4-mut) were induced with Dox for three hrs (+E4orf4)) or were left uninduced (-E4orf4), and were then treated with 4 μM hydroxyurea (HU) or with 25 ng per ml neocarzinostatin (NCS), or were left untreated (Control). Protein extracts were prepared after 3 hrs incubation with the drugs and subjected to Western blot analysis, using antibodies to phosphorylated and non-phosphorylated Chk1 and to E4orf4. Quantitation and normalization were performed as described above.

An interaction of E4orf4 with PP2A, mediated by regulatory B subunits, has been reported to contribute to all E4orf4 functions known to date [43, 47, 49, 60, 62–64]. Since E4orf4 appeared to reduce DDR protein phosphorylation (Fig 1), we examined whether this function required the E4orf4-PP2A interaction. Two experimental systems were examined: virus infection of cells in which the PP2A-B55 subunit could be inducibly knocked down, or drug treatment of cells expressing WT E4orf4 or a mutant that cannot bind PP2A. In the first approach, dl366* and dl366*+E4orf4 mutant viruses were used to infect the L11 cell line in which a PP2A-B55 shRNA can be transiently expressed by doxycycline (Dox) induction. Protein extracts were prepared 24 hrs post-infection and were subjected to Western blot analysis. Similarly to our previous observations in HeLa and HUVEC cells (Fig 1), infection of L11 cells with dl366* in the absence of Dox resulted in DDR activation manifested by enhanced phosphorylation of three sites in the Chk1 protein (S317, S345, and S296) that were barely phosphorylated in mock-infected cells. Infection with a similar MOI of dl366*+E4orf4 resulted in decreased phosphorylation of these sites without a similar reduction in Chk1 protein levels (Fig 2A, lanes 1–3). However, when Dox was added to the cells 72 hrs before infection, PP2A-B55 protein levels were dramatically reduced, and Chk1 phosphorylation was not affected by E4orf4 (Fig 2A, lanes 4–6). To ascertain that the reduced effect of E4orf4 on DDR activation was indeed the result of PP2A-B55 knockdown, a plasmid encoding HA-tagged PP2A-B55 which contained silent mutations rendering it resistant to the shRNA was transfected into control and Dox-treated L11 cells one day before infection. In the presence of exogenously expressed PP2A-B55, E4orf4 reduced Chk1 phosphorylation much better both in the presence and absence of Dox (Fig 2A, lanes 7–12), indicating that PP2A-B55 knockdown was indeed the cause of the diminished ability of E4orf4 to reduce Chk1 phosphorylation. Because the HEK293-derived L11 cells contain endogenous E1A and E1B proteins, E1B-55K staining could not be used in these cells to test the similarity of infection. However, a parallel infection of W162 cells with the same MOI revealed similar levels of E1B-55K protein in both dl366* and dl366*+E4orf4 virus infections (S1 Fig), confirming the uniformity of infection. The effect of E4orf4 on ATM phosphorylation was very small in the HEK293-derived L11 cells, and was therefore not studied in these cells.

An additional approach was utilized to confirm the findings indicating that E4orf4 must bind PP2A to reduce DDR protein phosphorylation. Cells containing Dox-inducible WT E4orf4 (clone 13) or the R81F84A E4orf4 mutant that does not bind PP2A (clone 3) were induced with Dox for three hrs, or were left uninduced, and were then treated with hydroxyurea (HU), which induces replication stress or with neocarzinostatin (NCS), a drug that induces DSBs. Protein extracts were prepared and subjected to Western blot analysis. As seen in Fig 2B, HU or NCS treatment caused Chk1 phosphorylation in both clone 13 (lanes 3,5) and clone 3 cells (lanes 9,11). However, expression of WT E4orf4 led to reduced Chk1 phosphorylation in clone 13 cells, especially on S317, and following NCS treatment on S296 (lanes 4,6) whereas in clone 3 cells expression of the R81F84A mutant did not alter Chk1 phosphorylation on S317 and altered S296 phosphorylation much less efficiently (lanes 10,12). The dissimilarities in DDR activation in the presence of the WT and mutant E4orf4 proteins did not result from different expression levels of these proteins. Thus the results support the conclusion that the interaction between E4orf4 and PP2A is required for the reduction in DDR protein phosphorylation which accompanies E4orf4 expression. The results also indicate that E4orf4 can inhibit the response to DNA damage outside the context of virus infection.

### E4orf4 inhibits DNA damage repair

**Fig 3:**
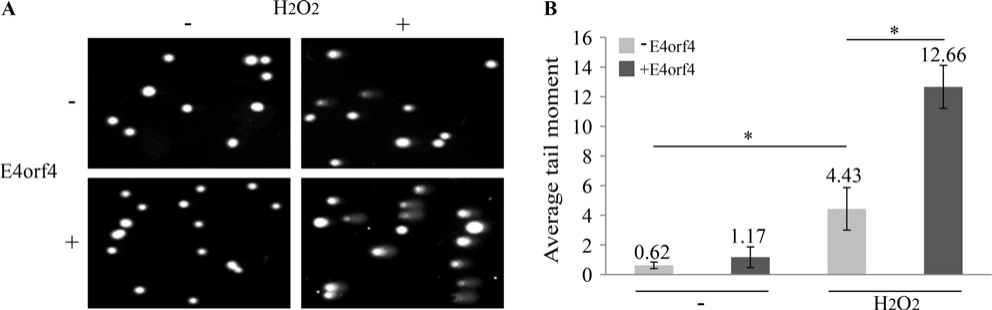
E4orf4 inhibits DNA damage repair. (**A**) Clone 13 cells were induced with Dox for 2.5 hrs to stimulate E4orf4 expression (+E4orf4), or were left uninduced (-E4orf4), and were then treated with 100 μM H_2_O_2_ for 30 minutes or were left untreated. Cell samples were then subjected to an alkaline comet assay. Representative microscopic images of the assay taken at a magnification of 200 are shown. (**B**). Quantitation of the comet data from A is shown. The length and intensity of DNA tails relative to heads is represented by the comet tail moment which was determined using the TriTek Comet Score software (n ≥ 100). Bars represent standard error of the mean. The statistical differences between control and E4orf4-expressing cells or between drug-treated and untreated cells are indicated with p values: *: p<0.001.

Based on our findings that E4orf4 obstructed DNA damage signaling, we expected E4orf4 to inhibit DNA damage repair. A direct evidence of a repair defect in E4orf4-expressing cells was obtained using the alkaline comet assay, which monitors the presence of DNA damage in single cells by microscopic detection of DNA migration in gel [67]. Clone 13 cells induced by Dox for 2.5 hrs to stimulate E4orf4 expression or uninduced cells were treated with H_2_O_2_ for 30 min or were left untreated and average comet tail moment was determined. The results demonstrate a significant increase in the number of comets seen in H_2_O_2_-treated cells in the presence of E4orf4, indicating a defect in damage repair in cells expressing the viral protein (Fig 3).

### ATM and ATR are not mutually required for inhibition of their signaling pathways by E4orf4

**Fig 4:**
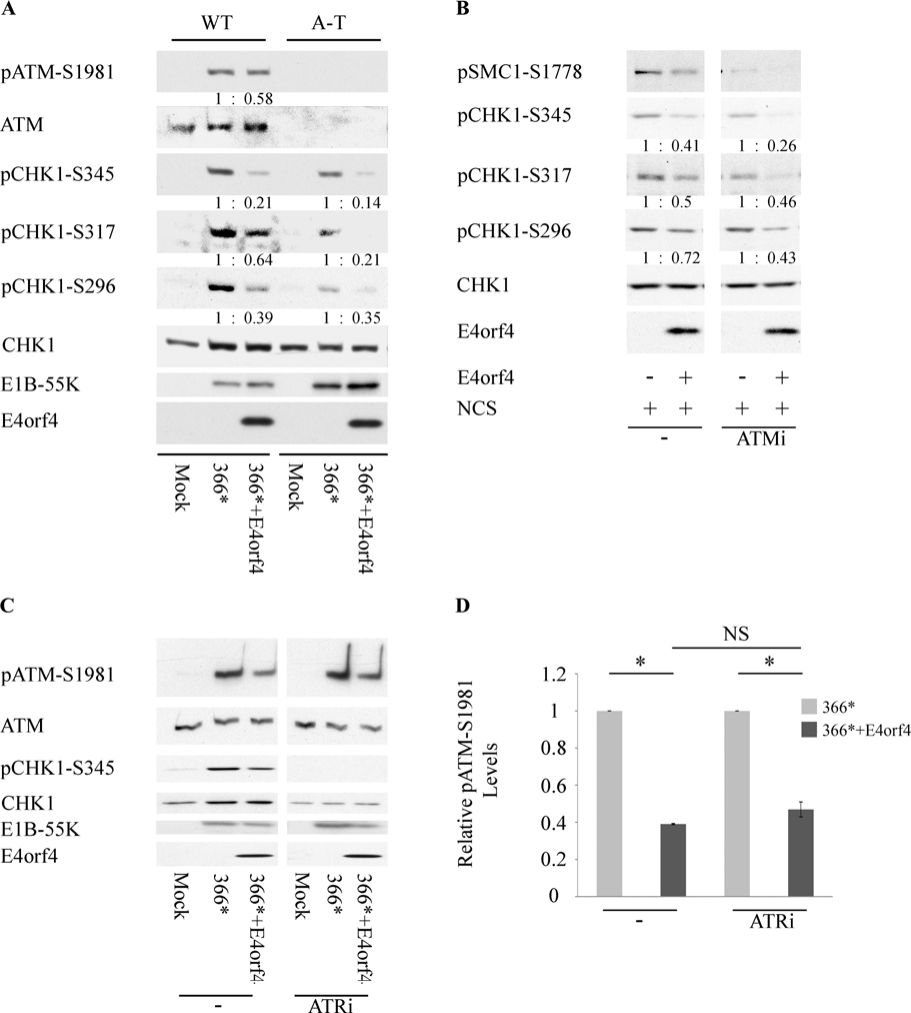
ATM and ATR are not mutually required for inhibition of their signaling pathways by E4orf4. (**A**) ATM-deficient A-T fibroblasts and matching WT fibroblasts were either mock-infected or infected with the Ad mutants dl366* and dl366*+E4orf4. Proteins were harvested 24 hrs later and Western blot analysis was carried out with the indicated antibodies. Quantitation and normalization were performed as described in the legend to Fig 1. (**B**) Clone 13 cells expressing E4orf4 under Dox regulation were induced for 3 hrs (+) or were left uninduced (-). They were then treated with NCS for one hr in the presence or absence of an ATM inhibitor (ATMi: KU60019). Protein extracts were subjected to Western blot analysis with the indicated antibodies. Quantitation and normalization were performed as described in the legend to Fig 1. (**C**) HeLa cells were either mock-infected or infected with the Ad mutants dl366* and dl366*+E4orf4. An ATR inhibitor (ATRi: ETP46464) was added to the infected cells and another group of infected cells was left untreated (-). Proteins were harvested 24 hrs post infection and Western blot analysis was carried out with the indicated antibodies. (**D**) The intensities of protein bands in Western blots from two independent experiments performed as described in C were quantified by densitometry. Relative pATM-S1981 levels were calculated as described in the legend to Fig 1, and the average of the two experiments is shown in the graph. Error bars represent standard error of the mean and statistical significance was determined using a paired student t test. *: p<0.003; NS: non-significant (p = 0.09).

Because E4orf4 inhibited phosphorylation of both ATM and ATR targets (Fig 1), and as it was reported that ATM and ATR signaling were not dependent on each other during Ad infection [38], we set out to determine whether E4orf4 reduced phosphorylation of ATM and ATR targets independently of each other, using mutant cells or kinase inhibitors. A-T cells, lacking ATM, and the corresponding WT cells were infected with dl366* or with dl366*+E4orf4. As seen in Fig 4A, activation of both ATM and Chk1 phosphorylation occurred in WT cells infected with dl366*, whereas upon infection with dl366*+E4orf4, ATM phosphorylation at S1981 and Chk1 phosphorylation at three phospho sites were reduced. In A-T cells, ATM was indeed missing, as expected. Phosphorylation of the ATR substrate Chk1 was induced in these cells by dl366* at slightly lower levels compared to WT cells. This may result from the somewhat reduced Chk1 levels in the infected A-T samples, or from loss of a partial indirect contribution of the ATM pathway to ATR activation through end resection [68, 69]. However, a reduction in phosphorylation of all tested Chk1 phospho sites was observed upon infection with dl366*+E4orf4, suggesting that ATM was not required for the reduced phosphorylation of ATR substrates induced by E4orf4. To further confirm this result, clone 13 cells were either induced for E4orf4 expression or left uninduced and were then treated with NCS in the presence or absence of an ATM inhibitor (KU60019). Fig 4B demonstrates that the ATM inhibitor, which diminished phosphorylation of the ATM substrate Smc1-S1778, did not prevent E4orf4 from reducing Chk1 phosphorylation. Thus the results indicate that reducing the phosphorylation of ATR substrates by E4orf4 does not require ATM. Interestingly, E1B-55K levels in A-T cells infected with both dl366* and dl366*+E4orf4 viruses were higher than in WT cells (Fig 4A), suggesting that ATM deficiency may improve virus replication, as discussed below.

To investigate the contribution of ATR to inhibition of DNA damage signaling by E4orf4, HeLa cells were infected with the dl366* and dl366*+E4orf4 mutant Ad viruses in the presence or absence of an ATR inhibitor (ETP46464, [70]). Addition of the ATR inhibitor to the infected cells for 22 hrs did not diminish ATM phosphorylation while resulting in complete loss of Chk1 phosphorylation. When the ability of E4orf4 to reduce ATM phosphorylation was compared in the presence or absence of the ATR inhibitor, no significant changes were observed and a 2.1–2.6 fold reduction occurred in both cases (Fig 4C and 4D). Thus, inhibition of ATR did not diminish the ability of E4orf4 to reduce ATM phosphorylation. Taken together the results indicate that the ATM and ATR signaling pathways can be inhibited by E4orf4 independently of each other.

### E4orf4 expression and ATM or ATR deficiency increase the efficiency of Ad replication

**Fig 5:**
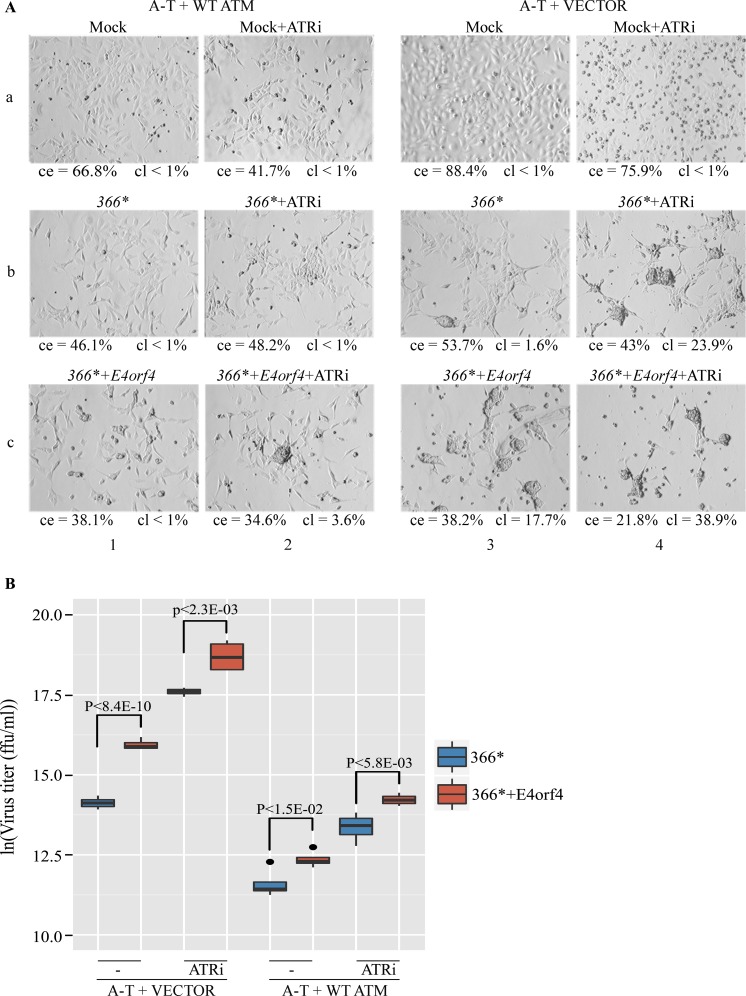
ATM and ATR inhibit Ad replication whereas E4orf4 enhances it. (**A**) ATM-deficient A-T cells, reconstituted with WT ATM or with an empty vector, were infected with the Ad mutants dl366* and dl366*+E4orf4 (30 ffu/cell), or were mock infected. An ATR inhibitor (ATRi) was added to parallel cell samples as described in the methods section. The cells were visualized 24 hrs post infection using a Zeiss Axioskop at a magnification of 100. Representative pictures of two independent experiments are shown. The percent of area covered with cells out of the total field area (ce) was determined using Image-Pro Premier 9.1 and the percent of area covered with cell clusters larger than 6000 pixels^2^ out of total cell area (cl), was calculated using FIJI. (B) Viruses from two independent experiments performed as described in A were collected from infected cells 24 hrs post infection and their titer was determined. Data is displayed in box plots as previously described [72] and black dots represent outliers. Statistical significance was calculated using unpaired t-test and p values of significant differences between dl366* and dl366*+E4orf4 viruses are shown.

**Fig 6:**
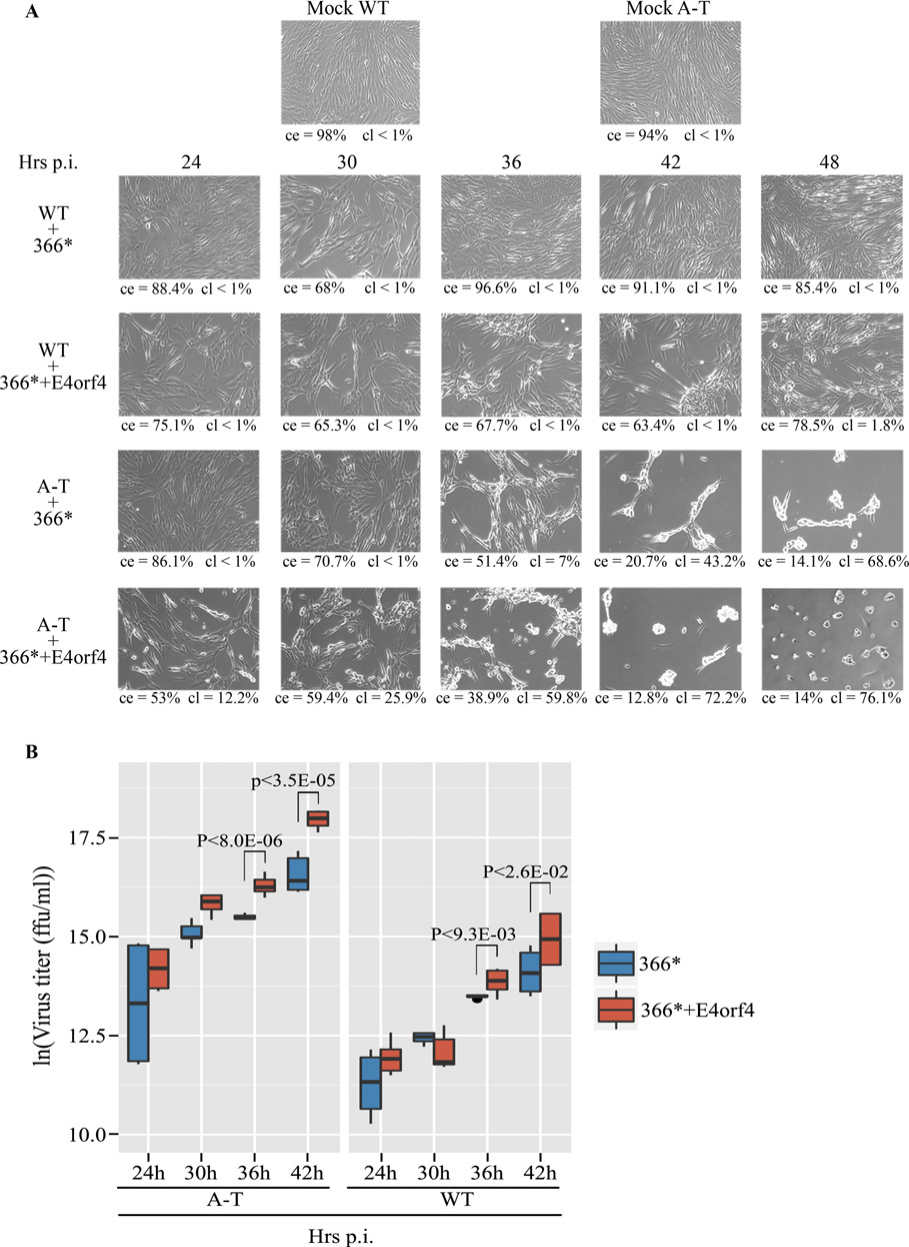
Loss of ATM and expression of E4orf4 accelerate Ad infection. (**A**) A-T cells and matching WT control cells were infected with the Ad mutants dl366* and dl366*+E4orf4 (100 ffu/cell) or were mock infected. The cells were visualized at the indicated times post infection (p.i.) using a Zeiss Axioskop at a magnification of 100. Mock infected samples are from the 24 hrs time point. Representative pictures of two independent experiments are shown. The percent of area covered with cells out of the total field area (ce) was determined using Image-Pro Premier 9.1 and the percent of area covered with cell clusters larger than 1000 pixels^2^ out of total cell area (cl), was calculated using FIJI. (**B**) Production of viruses in cells infected as described in A was determined by a titer assay. p.i.: post infection. Data is displayed in box plots as previously described [72]. Statistical significance was calculated using unpaired t-test and p values of significant differences between dl366* and dl366*+E4orf4 viruses are shown.

Since E4orf4 appears to inhibit the ATM- and ATR-regulated DDR (Figs 1, 2 and 4) and DDR was suggested to be detrimental to Ad infection, we examined the contribution of E4orf4 and the DDR components ATM and ATR to the efficiency of virus infection. ATM-deficient A-T cells reconstituted with an empty vector or a vector expressing WT ATM (S2 Fig, [71]) were infected with dl366* and dl366*+E4orf4 at 30 ffu/cell, in the presence or absence of an ATR inhibitor. Morphological changes in the cells and production of infectious viruses were assayed 24 hrs after infection. The cytopathic effects (CPE) that accompany Ad infection, including aggregation, rounding up, and detachment of cells were quantified in two ways. First, the ratio of area covered with adherent cells to the total area was determined (ce), indicating changes in cell number or a clustering of cells. Second, the ratio of area covered with cell clusters larger than a predetermined size to total cell area was calculated (cl), indicating the appearance of large cell clusters. As seen in Fig 5A, CPE were detected more clearly during dl366* infection of A-T cells reconstituted with the empty vector, which lack ATM, than in A-T cells expressing WT ATM (Fig 5A, compare squares b3 and b1). Moreover, the presence of E4orf4 significantly increased the CPE in A-T cells lacking ATM (Fig 5A, compare b3 and c3) and slightly increased CPE in A-T cells expressing ATM (Fig 5A, compare b1 and c1). Addition of an ATR inhibitor dramatically increased the CPE in dl366*- as well as in dl366*+E4orf4-infected cells lacking ATM (Fig 5A, b4 and c4) and somewhat increased CPE in dl366*+E4orf4-infected cells expressing ATM (Fig 5A, c2). However, during infection with dl366*, no difference in CPE was observed in A-T cells reconstituted with WT ATM and treated with the ATR inhibitor compared with no ATRi treatment (Fig 5A, b1 and b2). Efficiency of virus replication was quantified by collecting viruses generated in the cell samples shown in Fig 5A and assaying virus titers. The graph shown in Fig 5B and S1 Table illustrate numerically the enhanced efficiency of virus propagation in the absence of active ATM and ATR and in cells infected with dl366*+E4orf4 viruses compared with dl366* viruses. Thus, ATM deficiency as well as inhibition of ATR increased production of dl366* virus (10.5-fold (ln(fold change) = 2.44) and 5.68-fold (ln(fold change) = 1.76) respectively) and the combination of both led to more than 270-fold increase in dl366* virus propagation (ln(fold change) = 6). Addition of E4orf4 to the E4-deficient virus further enhanced virus multiplication. The temporal progression of infection with dl366* and dl366*+E4orf4 mutant viruses in A-T cells versus matching WT cells was also examined and is demonstrated in Fig 6. CPE was observed earliest in A-T cells infected with dl366*+E4orf4 viruses (24 hrs), and then gradually appeared in A-T cells infected with dl366* and in WT cells infected with dl366*+E4orf4. No CPE was observed in WT cells infected with dl366* viruses up to 48 hrs p.i. (Fig 6A). Virus infection appeared to progress slower in these cells compared with the A-T cells reconstituted with WT ATM or the empty vector described in Fig 5. Virus propagation was measured by a titer assay and the graph shown in Fig 6B together with S2 Table illustrate the enhanced efficiency of virus infection in the absence of ATM or in the presence of E4orf4. Thus at 42 hrs post infection, dl366* produced 11-fold more progeny virus in A-T cells than in WT cells (ln(fold change = 2.46) and dl366*+E4orf4 produced 17-fold more progeny virus (ln(fold change = 3), indicating that ATM inhibits virus replication as was recently reported [40, 78]. Furthermore, dl366*+E4orf4 produced significantly more progeny virus than dl366* both in WT and in A-T cells at late times of infection (2.44-fold and 3.73-fold change respectively (ln(fold change) = 0.83 and 1.39)). Taken together, the results indicate that deficiency in both ATM- and ATR-regulated DDR as well as expression of E4orf4, which inhibits both these DDR pathways, led to a more efficient progression of Ad replication.

**Fig 7:**
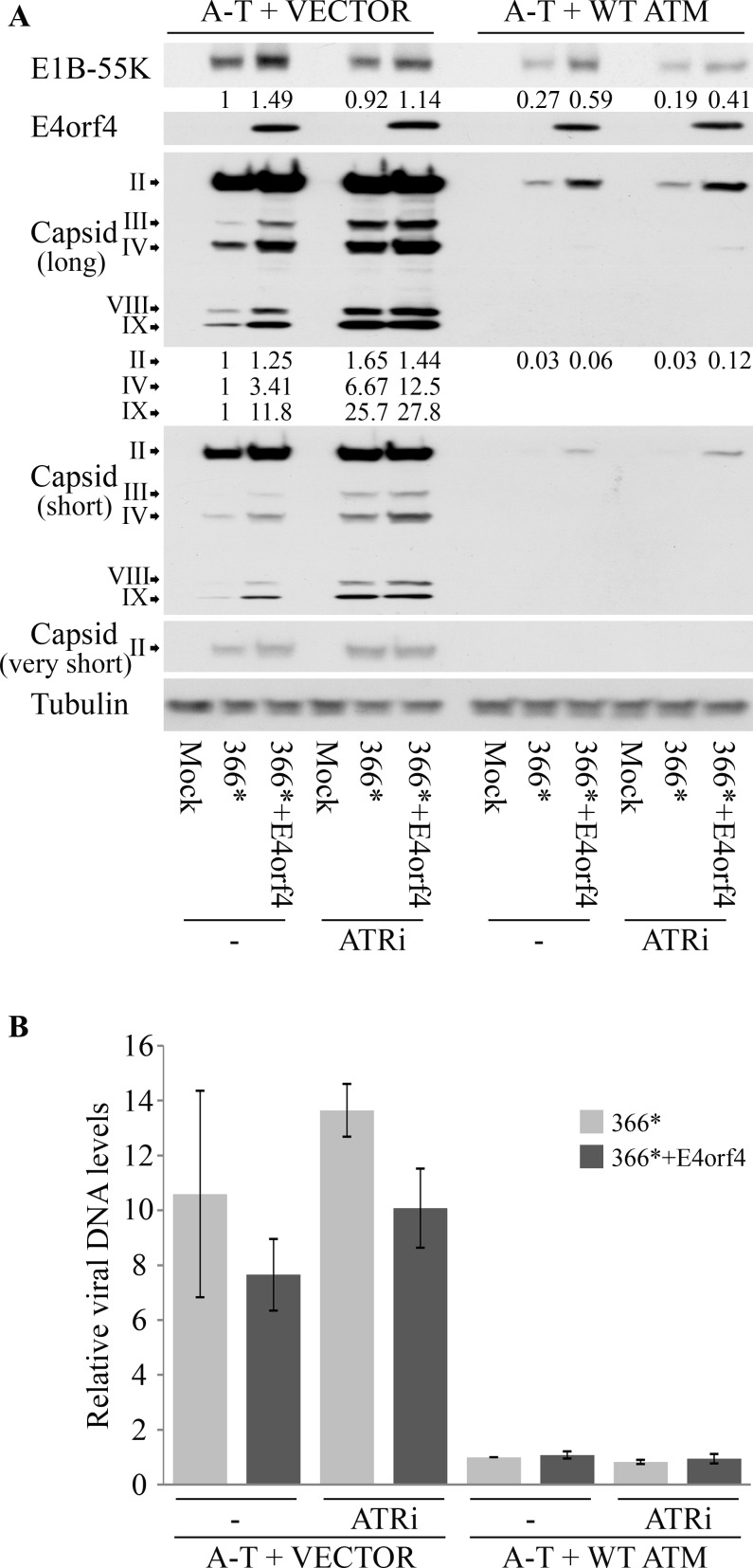
The effect of ATM and ATR inactivation and E4orf4 expression on various stages of Ad replication. (**A**) ATM-deficient A-T cells, reconstituted with WT ATM or with an empty vector, were infected with the Ad mutants dl366* and dl366*+E4orf4, or were mock infected. An ATR inhibitor (ATRi) was added to parallel cell samples as described in the methods section. Proteins were harvested 24 hrs later and Western blot analysis was carried out with the indicated antibodies. Three different exposures of the blot stained with a capsid-specific antibody are shown (long, short, very short). Protein levels were quantified by densitometry. Values of virus protein levels obtained by densitometry were normalized to the Tubulin loading control. The value for dl366* infection of A-T + VECTOR cells was defined as 1 and relative protein levels are shown beneath the relevant protein bands. (**B**) Virus DNA levels were quantified by quantitative PCR and normalized to cellular DNA represented by the *PRPH2* gene. The viral DNA level in a dl366* infection of A-T + WT ATM cells was defined as 1 and relative DNA levels are shown.

Because the results described above suggest that ATM and ATR inhibit Ad propagation and that E4orf4 acts to antagonize their effects, we set out to determine which stages in viral infection are affected by these activities. ATM-deficient A-T cells reconstituted with an empty vector or a vector expressing WT ATM were infected with similar MOIs of dl366* and dl366*+E4orf4, in the presence or absence of an ATR inhibitor (ATRi), as described above (Fig 5). Protein extracts were prepared 24 hrs post-infection and subjected to Western blot analysis. Protein bands from appropriate blot exposures were quantified by densitometry and normalized to Tubulin levels. The normalized protein levels in A-T + VECTOR cells infected with dl366* were defined as 1 and relative levels of E1B-55K and capsid proteins pII, pIV, and pIX in the different infections are shown. As demonstrated in Fig 7, expression levels of E1B-55K were somewhat elevated in the absence of ATM, but ATR inhibition did not increase E1B-55K levels (Fig 7A). E4orf4 levels were altered similarly to E1B-55K (Fig 7A). In contrast, the levels of Ad capsid proteins were dramatically increased when ATM was absent and ATR inhibition further elevated the levels of most capsid proteins except hexon (pII). When both ATM and ATR were deficient, E4orf4 could still increase the levels of some capsid proteins but not of others (compare fiber (pIV) and pIX for example). E4orf4 increased hexon (pII) levels in WT cells (A-T + WT ATM), regardless of ATR activity. Viral DNA levels were also dramatically higher in the absence of ATM than in WT cells but ATR inactivation or E4orf4 expression did not appear to affect them significantly (Fig 7B). These results indicate that ATM inhibition in the cells used here was very important to the virus life cycle starting at the stage of viral DNA replication, whereas ATR inhibition impacted mostly late protein expression and progeny virus production and did not affect earlier stages of the Ad life cycle. Under the experimental conditions used here, the contribution of ATR inhibition to late protein expression was more obvious when ATM was absent, consistent with the changes in virus titer shown in Fig 5.

### E4orf4 sensitizes cells to killing by drugs inducing replication stress or DSBs

**Fig 8:**
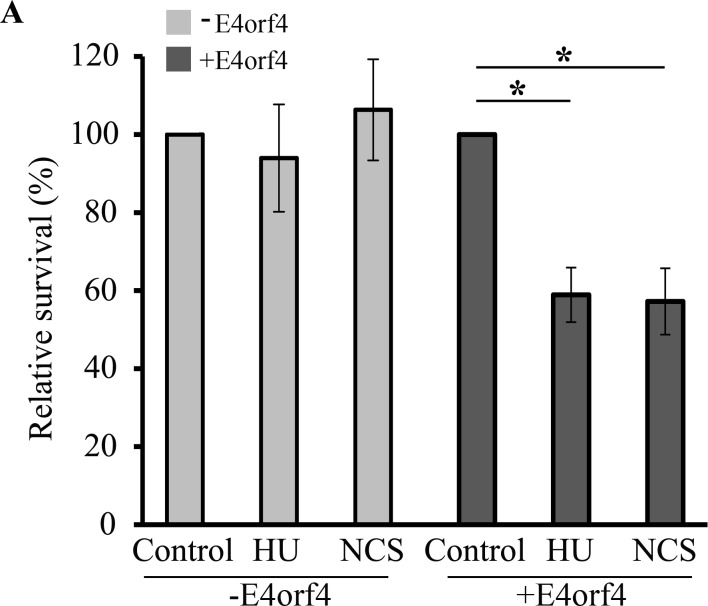
E4orf4 sensitizes cells to killing by drugs inducing DNA damage or replication stress. Clone 13 cells were induced with Dox for two hrs to stimulate E4orf4 expression (+E4orf4), or were left uninduced (-E4orf4), and were then treated with 4 μM HU or 2.5 ng/ml NCS for three hrs or left untreated (Control). Cell survival was measured by a clonogenic assay using various cell dilutions, without further Dox addition. The number of colonies was counted two weeks later, and relative survival was calculated separately for–E4orf4 and +E4orf4 samples by defining both control samples as 100%. E4orf4 itself decreased cell viability to 40% of control cells. Two independent experiments, each with triplicate samples were performed. Error bars represent pooled standard deviation and statistical significance was determined using a paired student t test. *: p<0.05.

Since E4orf4 reduced DDR activation and inhibited DNA repair, we examined whether these events resulted in sensitization of E4orf4-expressing cells to death induced by DNA damaging drugs. Clone 13 cells were induced by Dox for two hrs to stimulate E4orf4 expression or were left uninduced, and were then treated with 4 μM HU or 2.5 ng/ml NCS for three hrs or were left untreated. Cell survival was measured using a clonogenic assay at various cell dilutions, without further Dox addition. The number of colonies was counted two weeks later, and relative survival was calculated. As seen in Fig 8, the concentrations of HU and NCS used in these experiments were sub-lethal and did not reduce cell viability by themselves. However, when drug treatment was performed in combination with E4orf4 expression, cell viability was significantly reduced to less than 60% of the viability of E4orf4-expressing cells. These results suggest that E4orf4 sensitized the cells to treatment with genotoxic drugs or drugs causing replication stress.

## Discussion

### A novel mechanism for DDR inhibition by Ad

It has been reported that Ad utilizes several mechanisms to inhibit the DDR. The Ad E1B-55K and E4orf6 proteins cause degradation of various DDR proteins and E4orf3 expression leads to removal of the MRN DNA damage sensor complex from viral replication centers [33, 38, 39]. Moreover, while Ad was shown to induce parylation, the intracellular distribution of PAR-modified proteins was altered by the E1B-55K and E4orf3 proteins, possibly resulting in further modulation of the DDR [73]. The virus also utilizes its core protein VII to protect the incoming genome from the DDR [41]. The exploitation of several mechanisms for DDR attenuation by Ad suggests the importance of this process to virus replication. Our current work demonstrates that the Ad E4orf4 protein contributes a novel mechanism for DDR inhibition. In collaboration with its major cellular partner, PP2A, E4orf4 reduced phosphorylation of several DDR proteins belonging to both the ATM- and ATR-regulated pathways. This inhibition of DNA damage signaling occurred during virus infection as well as when E4orf4 was expressed alone, and was observed both in transformed and non-transformed cells (Figs 1 and 2). E4orf4 reduced phosphorylation of DDR proteins without affecting total levels of the MRN component Nbs1 (Fig 1) which is known to be degraded when the Ad E1B-55K and E4orf6 proteins are present [6, 31]. Disruption of the DDR by E4orf4 resulted in increased accumulation of DNA damage, as demonstrated by a comet assay (Fig 3). This assay was performed in HEK293-derived clone 13 cells which contain the Ad E1 proteins and therefore we cannot rule out some involvement of E1A or E1B in the observed inhibition of DNA repair. However, E4orf4 induced a significant inhibition of repair in this genetic background and thus its role in this process must be critical. Furthermore, E4orf4 inhibited DNA damage signaling in various types of cells, including non-transformed cells, suggesting that it has an important role in inhibition of DNA damage repair. Our results further demonstrate that ATM and ATR are not acting downstream of each other in the signaling pathway initiated by E4orf4 to reduce their activity (Fig 4). PP2A was reported to have a profound impact on the DDR by regulating activity of the primary (ATM, ATR and DNA-PK) and secondary (CHK1 and CHK2) kinases involved in the signaling cascade. It also dephosphorylates downstream targets, such as γ-H2AX and broadly influences repair of DSBs [74]. Thus it is possible that E4orf4 targets the ATM and ATR branches of the DDR separately, by recruiting PP2A to different upstream regulators. Alternatively, it is possible that E4orf4 recruits PP2A to one upstream regulator that affects both these DDR branches. Potential examples of E4orf4-PP2A targets that could affect both ATM- and ATR-regulated pathways include the MRN complex which accumulates at DNA damage foci and initiates signaling involving activation of ATM and ATR kinases [6, 8, 75], PARP-1 which interacts with both ATM and ATR and parylates them [76, 77], or other upstream DDR regulators. Future research will address the identification of direct targets of the E4orf4-PP2A complex in the DDR.

### E4orf4 expression and inhibition of ATM and ATR contribute to the efficiency of Ad replication

Our results indicate that ATM and ATR inhibition, as well as E4orf4 expression, increase the efficiency of Ad replication to various extents (Figs 5–7). In A-T+WT ATM cells, E4orf4 enhanced virus progeny production only slightly (2-fold, ln(fold change) = 0.76), and we could not detect an E4orf4 effect on viral DNA replication in these cells whereas E4orf4 did increase early and late protein expression to some extent (Fig 7). Because ATM deficiency increased Ad DNA replication (Fig 7B), these results could possibly reflect a low ability of E4orf4 to inhibit ATM activity in the A-T+WT ATM cells. The finding that E4orf4 could enhance progeny virus production without increasing viral DNA levels may reflect the possibility that DNA levels are not the limiting factor in Ad propagation in these cells and therefore the increase in late protein expression is responsible for generation of more infectious viruses. It is also theoretically possible that E4orf4 does not increase total virus DNA levels, but increases the amount of functional DNA, for example by reducing concatenation. However, it was previously reported that ATM and ATR knockdown did not affect mutant virus DNA concatenation in HeLa cells [78]. ATM deficiency led to a 10.5-fold increase in dl366* replication and ATR inactivation increased it by 5.7-fold (Fig 5 and S1 Table). When both ATM and ATR were deficient, the efficiency of dl366* infection was increased by 271-fold (ln(fold) = 6) and E4orf4 further increased replication efficiency by close to 3.3-fold (ln(fold) = 1.11). These results suggest that inhibition of both ATM and ATR by Ad proteins contributes to the efficiency of virus replication. However, besides its contribution to ATM and ATR inhibition, E4orf4 has additional functions that are beneficial for virus replication, such as down-regulation of early gene expression, regulation of splicing of viral RNAs and control of protein translation [44–49]. Execution of these functions may account for the enhancement of the efficiency of virus replication when both ATM and ATR are inhibited. It is also possible that E4orf4 inhibits additional DDR pathways.

It was previously reported that ATM interfered with DNA replication and late protein expression of an E4 mutant Ad in HeLa cells, whereas ATR was not found to interfere with late protein expression in these cells [78]. ATR inhibition was similarly not found to affect replication of a virus lacking E1B-55K and E4orf3 in A549 cells, whereas ATM inhibition significantly increased replication of this virus [40]. Another report presented results showing that neither ATM nor ATR depletion contributed to mutant Ad DNA replication in various cell lines derived from HeLa, U2OS and A-T cells [79]. The reasons for the inconsistent results are not known, but may include the use of different types of cells and different Ad E4 mutants (dl366* [65] vs. *dl1004* or *dl1007* [80], and ΔE1B-55K-ΔE4orf3 [40]). The significant effect of the ATR inhibitor used here on virus titer (Fig 5) was accompanied by abolishment of Chk1 phosphorylation while no effect on ATM phosphorylation was observed (Fig 4). This inhibitor was also reported to be deficient in inhibiting DNA-PK autophosphorylation [70], indicating its specificity. We found the effect of ATR inhibition to be more prominent when ATM was also absent (Fig 5 and S1 Table) and it may thus be easier to detect under these conditions. Interestingly, whereas ATM inhibition contributed to progression of the Ad replication cycle starting at the DNA replication stage, ATR appeared to exert its effect only later, at the time of late protein expression (Fig 7). We also observed that ATM activation by dl366* infection of HeLa cells occurred earlier than ATR activation (represented by Chk1 phosphorylation) (Fig 1), suggesting that ATR is activated and could exert its effect only at the later stages of infection. The mechanisms by which ATR can inhibit late Ad protein expression are not known, but may include an effect on alternative splicing [81] that could critically impact late gene expression. E4orf4 is known to regulate some alternative splicing events of viral mRNAs [48] and may work, at least in part, via inactivation of ATR. E4orf4 increased fiber protein (pIV) levels both in the presence and absence of an active ATR, whereas it enhanced pIX levels when an active ATR was present but not when ATR was inhibited. These findings could indicate different mechanisms of E4orf4-mediated effects on late protein expression, some of which may depend on ATR inhibition. Our results together with the lengths to which Ad goes to inhibit ATM and ATR signaling, strongly suggest that both ATM and ATR can inhibit Ad replication, at least under some conditions, and that their elimination is therefore important for efficient virus infection.

### Inhibition of the DDR and cancer-specific E4orf4-induced cell death

The results presented here indicate that E4orf4 increases the accumulation of DNA damage following treatment of cells with DNA damaging drugs (Fig 3), resulting in sensitization of the cells to killing induced by sub-lethal concentrations of DNA damaging drugs and drugs inducing replication stress (Fig 8). Because the experiments presented in Fig 8 were performed in cells derived from HEK293 cells which express the Ad E1A and E1B proteins and these proteins have their own effect on cell survival, we cannot rule out some influence of the E1A and E1B proteins. However, E4orf4 further increased the susceptibility of the cells to drug-induced cell death indicating that it has an important contribution to sensitization of these cells to DNA damaging drugs.

It has been reported that when E4orf4 is expressed alone, it induces a unique mode of cancer-specific cell death [60, 61]. Inhibition of the DDR may contribute to this process. Previous reports have indicated that deficiencies in DDR mechanisms are contributing factors in many stages of tumor development [82, 83]. Many malignant tumors show functional loss or deregulation of key proteins involved in the DDR, including p53, ATM, Mre11, BRCA1/2 and Smc1. Such mutations may allow pre-cancerous cells to evade the proliferation barrier created by the DDR, thus allowing the progression of pre-malignant lesions to full malignancy [84]. While defects in DDR components may confer a growth advantage on cancer cells, allowing them to survive and proliferate despite oncogene-induced replication stress and genomic instability, they may also cause cancer cells to rely on the remaining DDR pathways in order to survive DNA damage. Targeting of the remaining pathways by a DDR inhibitor such as E4orf4 may therefore be selectively toxic to cancer cells with mutations in DDR genes.

In summary, E4orf4 employs a novel mechanism to inhibit the DDR, which improves Ad replication and may contribute to induction of cancer-specific cell death by the viral protein. Investigation of this novel mechanism may provide a better understanding of critical DDR nodes that are targeted by E4orf4 and are required for successful application of a combinatorial treatment of cancer. Moreover, our results are important for the understanding of Ad-host cell interactions. The lengths to which Ad goes to inhibit the DDR indicate that DDR inhibition is central to the virus life cycle and our findings enhance this conclusion. Ads have been identified in recent years as significant pathogens in immunocompromised patients [85]. As there is no virus-specific therapy for Ad infection, it has become very important to understand the host responses to Ad infection and the viral strategies used to inhibit these responses in order to promote the development of antiviral therapies. Thus understanding the E4orf4 role in inhibition of the DDR may contribute to the development of new anti-viral and anti-cancer treatments.

## Materials and Methods

### Cells, plasmids, virus mutants and infections

HeLa cells (American Type Culture Collection) were cultured in Dulbecco's Modified Eagle's medium (DMEM) supplemented with 10% fetal calf serum (FCS). Clone 13 cells containing tetracycline-inducible E4orf4, clone 3 cells containing a tetracycline-inducible E4orf4 mutant that does not bind PP2A, and clone L11 cells containing a tetracycline-inducible PP2A-B55 shRNA were generated by introducing a tetracycline-inducible pSUPERIOR vector (OligoEngine) containing cDNAs for WT E4orf4 [43], the E4orf4 mutant R81F84A that does not bind PP2A [63], or a PP2A-B55alpha shRNA [86] into T-REx-293 Cells (Invitrogen by Life Technologies). The resulting cell lines were propagated in DMEM supplemented with 10% FCS guaranteed to be tetracycline-free (BD Bioscience), 5 μg/ml blasticidine (Invitrogen), and 200 μg/ml zeocin (Invitrogen). Doxycycline induction was done by addition of 1 μg/ml doxycycline, whereas uninduced cells received an equal amount of the solvent. A-T cells and matching WT fibroblasts were from Y. Shiloh (Tel Aviv University) and were grown in DMEM supplemented with 10% FCS. A-T cells reconstituted with empty vector (GM16666) or the vector expressing WT ATM (GM16667) were from the Coriell Institute and were grown in DMEM supplemented with 15% FCS and 100 μg/ml hygromycin. Human Umbilical Vein Endothelial Cells (HUVEC) were a gift from G. Neufeld and O. Kessler (Technion). These cells were cultured on gelatin-coated dishes in M-199 medium containing 20% FCS, 2 mM glutamine, antibiotics, and 2 ng/ml bFGF, which was added every other day to the cells [87].

A PP2A-B55alpha shRNA-expressing pSUPER plasmid was a gift from S. Strack [86] and the shRNA sequence was subcloned from this plasmid into a tetracycline-inducible pSUPERIOR vector (OligoEngine) for use in the generation of the L11 cell line. The PP2A-B55alpha mutant resistant to the PP2A-B55alpha shRNA was previously described [43].

Adenoviral mutants dl366*, lacking the complete E4 region, and dl366*+E4orf4, lacking all E4 open reading frames (orfs) except E4orf4, were previously described [65] and were propagated on W162 cells (from T. Shenk, Princeton University, [88]). W162 cells were also used for determination of virus titer. Virus infections were performed at a multiplicity of 20–30 fluorescent forming units (ffu) in medium supplemented with 2% FCS at 37°C for 2 hrs, after which additional serum was added to a total of 10%, 15%, or 20%, as required for the specific cell line. Kinase inhibitors were added to infected cells 2 hrs post infection at the following concentrations: the ATM inhibitor KU60019 (Tocris Bioscience): 5μM, the ATR inhibitor ETP46464 [70]: 1 μM. An identical ATM inhibitor quantity and 50% of the original ATR inhibitor were added again 9 hrs later. Untreated cells received equal quantities of the solvent. Infected cells were harvested for protein extraction or virus collection at the indicated times.

### Relative quantitation of viral DNA

Relative levels of viral DNA were monitored by quantitative PCR, as previously described [89]. Total infected cell extracts were prepared in RIPA buffer (150 mM NaCl, 50 mM Tris, pH 8.0, 1.0% IGEPAL CA-630, 0.5% sodium deoxycholate, 0.1% SDS), sonicated, and treated with proteinase K. Quantitative real-time PCR was performed using hexon-specific primers: hexon-qPCR-fw: 5′-CGCT GGACATGACTTTTGAG-3′; hexon-qPCR-rev: 5′-GAACGGTGTGCGCAGGTA-3′. Results were normalized to levels of the cellular *PRPH2* gene determined by a parallel quantitative PCR with the primers: TM684-fw: 5’-CTGAAGCCGTACCTGGCTATC-3’; TM685-rev: GTGTCCCGGTAGTACTTCATGC.

### Western blot analysis

Whole cell extracts were prepared in SDS sample buffer (62.5 mM Tris-HCl pH 6.8, 2% SDS, 50 mM DTT, 10% glycerol) and viscosity was reduced by passing the extracts several times through a 27G needle after three min incubation at 95°C. Proteins were analyzed by Western blots using the indicated antibodies. Blot images were scanned with an Epson Photo 4990 scanner and were processed using Adobe PhotoShop 5.0 or 7.0. Band intensities were quantified using the TotalLab software.

Antibodies specific for the following proteins were used in this work: E4orf4 [54], DBP (clone B6) [90], E1B-55K (clone 2A6) [91], PP2A-B55 [43], HA (Covance), pATM-S1981 and ATM (mouse) (Epitomics), pChk1- S296 or S317 or S345, pChk2-T68, p53BP1-S1778, pNbs1-S343 and Nbs1, pAkt-S473 and Akt (Cell signaling), pSmc1-S966 (Bethyl), ATM (rabbit), Chk1, Chk2 (Santa Cruz).

### Immunofluorescence

HeLa cells grown on glass coverslips were infected at a multiplicity of infection of 30 ffu/cell. After 24 hrs, the cells were washed, fixed, stained with the indicated antibodies and counter-stained with 4',6-diamidino-2-phenylindole (DAPI) (Sigma). Immunostaining was visualized using an Axiocam camera linked to a Zeiss Axioskop at the indicated magnification.

### Colony formation assay

Clone 13 cells were treated with Dox for 2 hrs to induce E4orf4 expression or were left uninduced and were then treated with hydroxyurea (HU) (Sigma, 4 μM) or neocarzinostatin (NCS) (Sigma, 2.5 ng per ml) for three hrs or left untreated. The cells were then harvested, counted, and plated in several decimal dilutions. Medium (without Dox) was changed every 3–4 days and colonies were counted after two weeks.

### Comet assay

Clone 13 cells were induced with Dox for 2.5 hours or were left uninduced and were then treated with 100 μM H_2_O_2_ for 30 minutes. An alkaline comet assay was performed as described before [92]. Briefly, cells were harvested and mixed with low-melting agarose. After lysis, the cells were incubated in an alkaline buffer for 30 min and subjected to electrophoresis performed at 1 V/cm for 30 min. The slides were then stained with ethidium bromide dye for 20 min, washed in PBS, dried, and images of at least 100 randomly selected cells per sample were captured by a Zeiss fluorescent microscope at a magnification of 200. Digital fluorescent images were obtained using the AxioVision software. In this assay, the length and intensity of DNA tails relative to heads is proportional to the amount of DNA damage in individual nuclei. These parameters were determined by measurements of comet tail moment using the TriTek Comet Score software (TriTek Corp., Sumerduck, VA).

### Statistical analysis

Box plots were generated using R software (version 3.1.2) and package ‘ggplot2’ (version 1.0.0). Data for box plot analysis was subjected to natural log transformation. Statistical significance was calculated using t-test.

## Supporting Information

S1 Fig Determination of uniformity of virus infection. W162 cells were infected with dl366* and dl366*+E4orf4 mutant viruses at 30 ffu/cell or were left uninfected (Mock). Protein extracts were prepared 24 hrs post-infection and E1B-55K proteins were detected by Western blot analysis. Alpha-Tubulin staining served as a loading control.
(TIF)

S2 Fig ATM and ATR expression in A-T cells reconstituted with an empty vector or with a vector expressing ATM. Expression of ATM and ATR in A-T cells reconstituted with an empty vector or with a vector expressing ATM was detected by Western blot analysis. Alpha-Tubulin staining served as a loading control.
(TIF)

S1 Table Virus titer data for Fig 5. Virus titers and ln(virus titers) from two independent experiments described in Fig 5 are shown. SE: Standard error.
(DOCX)

S2 Table Virus titer data for Fig 6. Virus titers and ln(virus titers) from two independent experiments described in Fig 6 are shown. SE: Standard error.
(DOCX)
