# Supplementary figures and images for: The Adenovirus E4orf4 Protein Provides a Novel Mechanism for Inhibition of the DNA Damage Response
Source: PLoS Pathog. 2016 Feb 11;12(2):e1005420. doi: 10.1371/journal.ppat.1005420 (PMC4750969; doi:10.1371/journal.ppat.1005420)

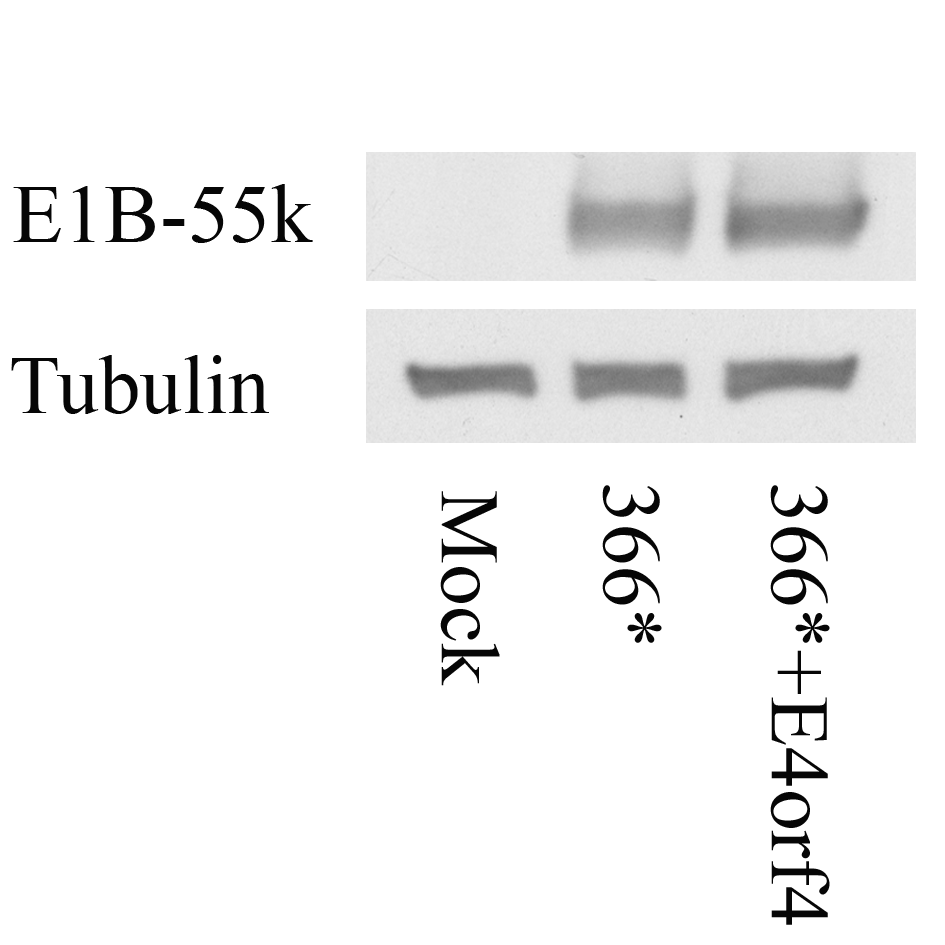

Supplement: S1 Fig — W162 cells were infected with dl366* and dl366*+E4orf4 mutant viruses at 30 ffu/cell or were left uninfected (Mock). Protein extracts were prepared 24 hrs post-infection and E1B-55K proteins were detected by Western blot analysis. Alpha-Tubulin staining served as a loading control. (TIF) [file ppat.1005420.s001.tif]

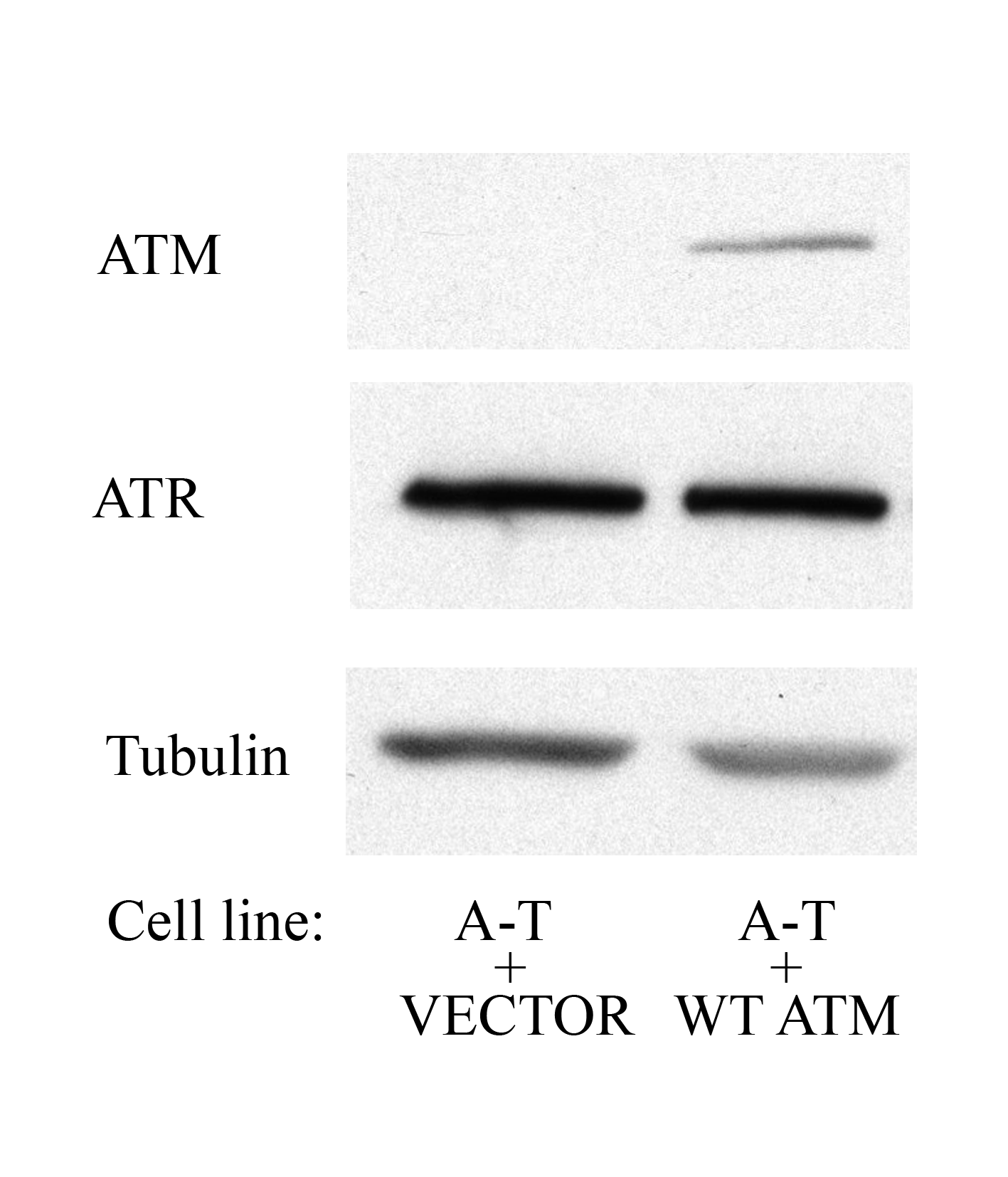

Supplement: S2 Fig — Expression of ATM and ATR in A-T cells reconstituted with an empty vector or with a vector expressing ATM was detected by Western blot analysis. Alpha-Tubulin staining served as a loading control. (TIF) [file ppat.1005420.s002.tif]
