# Supplementary material for: The Adenovirus E4orf4 Protein Provides a Novel Mechanism for Inhibition of the DNA Damage Response
Source: PLoS Pathog. 2016 Feb 11;12(2):e1005420. doi: 10.1371/journal.ppat.1005420 (PMC4750969; doi:10.1371/journal.ppat.1005420)
Supplement: S1 Table — Virus titers and ln(virus titers) from two independent experiments described in Fig 5 are shown. SE: Standard error. (DOCX) [file ppat.1005420.s003.docx]

Virus Titer (ffu/ml)

|  | **A-T + VECTOR** | | | | **A-T + WT ATM** | | | |
| --- | --- | --- | --- | --- | --- | --- | --- | --- |
|  | **-** | | ATRi | | **-** | | ATRi | |
|  | **366*** | **366***  **+E4orf4** | **366*** | **366***  **+E4orf4** | **366*** | **366***  **+E4orf4** | **366*** | **366***  **+E4orf4** |
| **Average** | 1.25E+06 | 7.66E+06 | 4.40E+07 | 1.45E+08 | 1.19E+05 | 2.40E+05 | 6.78E+05 | 1.52E+06 |
| **SE** | 8.85E+05 | 5.42E+06 | 3.11E+07 | 1.02E+08 | 8.44E+04 | 1.70E+05 | 4.79E+05 | 1.07E+06 |

Ln(Virus Titer(ffu/ml))

|  | **A-T + VECTOR** | | | | **A-T + WT ATM** | | | |
| --- | --- | --- | --- | --- | --- | --- | --- | --- |
|  | **-** | | ATRi | | **-** | | ATRi | |
|  | **366*** | **366***  **+E4orf4** | **366*** | **366***  **+E4orf4** | **366*** | **366***  **+E4orf4** | **366*** | **366***  **+E4orf4** |
| **Average** | 14.04 | 15.85 | 17.59 | 18.70 | 11.60 | 12.36 | 13.36 | 14.22 |
| **SE** | 0.07 | 0.03 | 0.09 | 0.35 | 0.33 | 0.19 | 0.32 | 0.13 |
