# Supplementary material for: The Adenovirus E4orf4 Protein Provides a Novel Mechanism for Inhibition of the DNA Damage Response
Source: PLoS Pathog. 2016 Feb 11;12(2):e1005420. doi: 10.1371/journal.ppat.1005420 (PMC4750969; doi:10.1371/journal.ppat.1005420)
Supplement: S2 Table — Virus titers and ln(virus titers) from two independent experiments described in Fig 6 are shown. SE: Standard error. (DOCX) [file ppat.1005420.s004.docx]

Virus Titer (ffu/ml)

|  | **A-T** | | | | | | | |
| --- | --- | --- | --- | --- | --- | --- | --- | --- |
|  | **24h** | | **30h** | | **36h** | | **42h** | |
|  | **366*** | **366***  **+E4orf4** | **366*** | **366***  **+E4orf4** | **366*** | **366***  **+E4orf4** | **366*** | **366***  **+E4orf4** |
| **Average** | 1.39E+06 | 1.62E+06 | 3.64E+06 | 7.69E+06 | 5.37E+06 | 1.21E+07 | 1.72E+07 | 6.41E+07 |
| **SE** | 9.71E+05 | 5.82E+05 | 7.52E+05 | 1.32E+06 | 2.56E+05 | 2.12E+06 | 6.02E+06 | 9.97E+06 |

|  | **WT** | | | | | | | |
| --- | --- | --- | --- | --- | --- | --- | --- | --- |
|  | **24h** | | **30h** | | **36h** | | **42h** | |
|  | **366*** | **366***  **+E4orf4** | **366*** | **366***  **+E4orf4** | **366*** | **366***  **+E4orf4** | **366*** | **366***  **+E4orf4** |
| **Average** | 8.60E+04 | 1.65E+05 | 2.55E+05 | 1.94E+05 | 7.22E+05 | 1.09E+06 | 1.53E+06 | 3.73E+06 |
| **SE** | 5.36E+04 | 5.10E+04 | 2.53E+04 | 6.98E+04 | 2.07E+04 | 2.38E+05 | 5.88E+05 | 1.65E+06 |

Ln(Virus Titer(ffu/ml))

|  | **A-T** | | | | | | | |
| --- | --- | --- | --- | --- | --- | --- | --- | --- |
|  | **24h** | | **30h** | | **36h** | | **42h** | |
|  | **366*** | **366***  **+E4orf4** | **366*** | **366***  **+E4orf4** | **366*** | **366***  **+E4orf4** | **366*** | **366***  **+E4orf4** |
| **Average** | 13.31 | 14.18 | 15.07 | 15.83 | 15.49 | 16.28 | 16.56 | 17.95 |
| **SE** | 1.14 | 0.39 | 0.20 | 0.18 | 0.05 | 0.17 | 0.33 | 0.16 |

|  | **WT** | | | | | | | |
| --- | --- | --- | --- | --- | --- | --- | --- | --- |
|  | **24h** | | **30h** | | **36h** | | **42h** | |
|  | **366*** | **366***  **+E4orf4** | **366*** | **366***  **+E4orf4** | **366*** | **366***  **+E4orf4** | **366*** | **366***  **+E4orf4** |
| **Average** | 11.00 | 11.94 | 12.44 | 12.08 | 13.49 | 13.86 | 14.11 | 14.93 |
| **SE** | 0.69 | 0.29 | 0.10 | 0.33 | 0.03 | 0.23 | 0.41 | 0.50 |
